# Activation of Cytotoxic and Regulatory Functions of NK Cells by Sindbis Viral Vectors

**DOI:** 10.1371/journal.pone.0020598

**Published:** 2011-06-02

**Authors:** Tomer Granot, Lisa Venticinque, Jen-Chieh Tseng, Daniel Meruelo

**Affiliations:** 1 Gene Therapy Center, Cancer Institute and Department of Pathology, New York University School of Medicine, New York, New York, United States of America; 2 Dana-Farber Cancer Institute, Boston, Massachusetts, United States of America; Centre de Recherche Public de la Santé (CRP-Santé), Luxembourg

## Abstract

Oncolytic viruses (OVs) represent a relatively novel anti-cancer modality. Like other new cancer treatments, effective OV therapy will likely require combination with conventional treatments. In order to design combinatorial treatments that work well together, a greater scrutiny of the mechanisms behind the individual treatments is needed. Sindbis virus (SV) based vectors have previously been shown to target and kill tumors in xenograft, syngeneic, and spontaneous mouse models. However, the effect of SV treatment on the immune system has not yet been studied. Here we used a variety of methods, including FACS analysis, cytotoxicity assays, cell depletion, imaging of tumor growth, cytokine blockade, and survival experiments, to study how SV therapy affects Natural Killer (NK) cell function in SCID mice bearing human ovarian carcinoma tumors. Surprisingly, we found that SV anti-cancer efficacy is largely NK cell-dependent. Furthermore, the enhanced therapeutic effect previously observed from Sin/IL12 vectors, which carry the gene for interleukin 12, is also NK cell dependent, but works through a separate IFNγ-dependent mechanism, which also induces the activation of peritoneal macrophages. These results demonstrate the multimodular nature of SV therapy, and open up new possibilities for potential synergistic or additive combinatorial therapies with other treatments.

## Introduction

Research into the utilization of oncolytic viruses (OVs) for cancer therapy has increased exponentially in the last few years. A variety of viruses have been used in these studies, including adenovirus, vaccinia virus, vesicular stomatitis virus, herpes simplex virus, and others (reviewed in [1]). OVs are unique in their ability to specifically kill tumor cells while sparing non-tumor cells, both *in vitro* and in various mouse models. Tumor specificity is achieved in different ways, including binding to receptors that are overexpressed in cancer cells, utilizing cancer-specific promoters, and taking advantage of defective anti-viral responses in tumor cells. Clinical studies have demonstrated OV safety, but the efficacy of OV therapy in human patients still needs to be determined.

In the first few years of OV research, much of the focus was on optimizing tumor targeting or viral replication in tumors. As a result, the immune response to the virus was seen merely as an obstacle to effective OV treatment. However, if the immune response to the virus could be redirected against the tumor, it could prove to be therapeutically beneficial [2]. Traditionally, cancer immunotherapy involved using specific immune modulators (e.g. cytokines or cells) to promote an anti-cancer immune response. As intracellular pathogens, viruses stimulate a variety of immune modulators and effector cells, which could also act against tumors. In addition, genetically engineered OVs can deliver specific immune-stimulating cytokines into the tumor site [3], thus limiting the side effects associated with systemic administration of cytokines [4], and potentially synergizing with virally induced cytokines.

Murine models for studying OV cancer therapy can be divided into two general groups: immunocompetent mice bearing syngeneic (spontaneous or implanted) tumors [5], and immunodeficient mice (typically SCID mice, which lack T and B cells, or nude mice, which lack T cells) implanted with human xenograft tumors [6], [7], [8]. An increasing number of studies using immunocompetent mouse models have demonstrated that immune cells (particularly NK cells and T cells) play an important role in the therapeutic effect induced by the virus [2], [9], [10]. However, far less is known about the role of immune cells in immunodeficient mouse models of OV therapy. Indeed, models using immunodeficient mice have often been considered to essentially lack immune cell activity, and as a result, effective OV treatment in these models was attributed to direct tumor killing. However, immunodeficient mice still retain important components of the innate immune response, including NK cells, macrophages, and neutrophils [11]. Given the role of immune cells in OV therapy in immunocompetent mice, the mechanism behind OV efficacy in immunodeficient mouse models should also be re-evaluated.

NK cells have evolved to recognize and kill abnormal host cells, including virally infected cells and tumor cells. In addition, activated NK cells can secrete cytokines that could influence the course of the immune response to a disease [12]. As such, they are uniquely interesting cells in the context of OV therapy, because they represent a “double bridge,” linking tumor cells to the more immunogenic OV-infected cells, while also linking the innate immune response to the potentially more potent adaptive immune response. One goal of OV therapy should therefore be to activate the cytotoxic and regulatory functions of NK cells against cancer.

Sindbis virus, an alphavirus with a positive single-stranded RNA genome, has several advantages that make it a good candidate for cancer therapy. In nature, SV does not cause severe disease in humans. Furthermore, the SV lifecycle does not have a DNA phase, so there is no risk of genomic integration. SV is believed to target tumors through interactions with the high-affinity laminin receptor [13], which is overexpressed in many cancers, though additional factors are likely also involved [14]. In our lab, we use a replication deficient form of SV, in which the structural proteins have been removed [15], further enhancing its safety. Replication defective SV vectors can also carry therapeutic genes into the tumor environment to generate a bystander therapeutic effect [16], in addition to inducing apoptosis in infected cancer cells [17]. Finally, because SV is a blood-borne pathogen, it can be injected systemically to target metastatic tumors [18]. Previous studies using SV vectors have demonstrated that they can target tumors, and have a strong therapeutic effect in various mouse models, including xenograft SCID models, syngeneic immunocompetent models, and a spontaneous tumor model [18]. Experiments using baby hamster kidney tumors in SCID/beige mice have pointed to a potential therapeutic role for NK cells [17]. Additionally, SV vectors carrying IL12, a known NK cell activator and immune modulator, have an enhanced therapeutic effect in SCID mice bearing human ovarian cancer tumors [18].

In this study, we show for the first time that SV vectors can activate NK cells in the peritoneum of SCID mice bearing intraperitoneally (i.p.) growing ES-2 cells, a human ovarian carcinoma cell line. Surprisingly, we also found that NK cell activation is critically important for effective SV treatment in this model. In addition, we found that the therapeutic effect of Sin/IL12, which further improves mouse survival, is dependent on both the cytotoxic and regulatory functions of NK cells. The regulatory function, which is dependent on IFNγ, also results in the upregulation of MHC class II on macrophages, demonstrating a shift towards the anti-cancer M1 phenotype. Taken together, our results show an important new mechanism for SV anti-cancer efficacy in SCID mice, with implications for future SV clinical studies and treatment designs.

## Materials and Methods

### Ethics Statement

All animal experiments were approved by the Institute of Animal Care and Use Committee at New York University Medical Center (protocol number 081115-03).

### Mice

6-12-week-old female C.B-17-SCID and C.B-17-SCID beige mice were purchased from Taconic (Germantown, NY). In some experiments, we used SCID mice from our own colony, which were generated by breeding C.B-17-SCID mice with *Ces1c^e^ Foxn1^nu^*/J mice (The Jackson Laboratory), and were selected for the absence of T and B cells, and for a deficiency in plasma esterase activity, which was needed for a separate study. No noticeable difference in NK cell activation was observed between SCID mice from our colony and C.B-17-SCID mice, and key experiments (FACS, NK depletion, etc.) were done at least once with C.B-17-SCID mice.

### Cell lines

Baby hamster kidney (BHK), ES-2 cells, and YAC-1 cells were obtained from the American Type Culture Collection. BHK cells were maintained in minimum essential α-modified media (αMEM, Mediatech, Inc.) with 10% fetal calf serum (FCS). ES-2 cells were cultured in McCoy's 5A medium (Mediatech) supplemented with 10% FCS. ES-2/Fluc cells (referred to as ES-2 cells here), which express firefly luciferase (Fluc), for noninvasive bioluminescent imaging, were derived from the ES-2 cell line by stable transfection of a plasmid, pIRES-2-Fluc/EGFP, as previously described [18]. YAC-1 cells were maintained in DMEM (Mediatech) containing 4.5 g/l glucose and supplemented with 10% FCS. All basal media were supplemented with 100 µg/mL of penicillin-streptomycin (Mediatech) and 0.5 µg/mL of amphotericin B (Mediatech).

### Sindbis Vector Production

Sindbis vectors (Sin/LacZ and Sin/IL12) were produced as previously described [17]. Briefly, plasmids carrying the replicon (SinRep-LacZ or SinRep-IL12) or DHBB helper RNAs were linearized with XhoI. *In vitro* transcription was performed using the mMessage mMachine RNA transcription kit (Ambion). Helper and replicon RNAs were then electroporated into BHK cells and incubated at 37°C in αMEM supplemented with 10% FCS. After 12 hours, the media was replaced with OPTI-MEM (GIBCO-BRL) supplemented with CaCl_2_ (100 mg/l) and cells were incubated at 37°C. After 24 hours, the supernatant was collected, centrifuged to remove cellular debris, and frozen at −80°C. Vectors were titered as previously described [17].

### FACS analysis

Anti-mouse antibodies (Abs) were purchased from ebioscience, San Diego, CA: Anti-CD45 PE-Cy7, anti-CD122 FITC, anti-CD335 (NKp46) FITC, anti-CD314 (NKG2D) PE, anti-CD178 (Fas Ligand) PE, anti-CD253 (TRAIL) PE, anti-CD49b (DX5)-PE anti-CD69 APC, anti-Ly-6C PerCP-Cy5.5, anti-Ly-6G (Gr-1) APC-eFluor® 780, anti-F4/80 Antigen PE, anti-MHC Class II (I-A/I-E) Alexa Fluor® 700.

For analysis of peritoneal cells with FACS, mice were euthanized, and their peritoneum was washed with 5 ml cold HBSS (Mediatech). Samples were then stained with various Abs, washed twice with HBSS, and analyzed using an LSR II machine (BD Biosciences, San Jose, CA). Data was analyzed using FlowJo (Tree Star, Inc.). NK cells were identified as CD122+ SSC (side scatter) low, or NKp46+ SSC low cells. Macrophages were identified as F4/80+ FSC (forward scatter) high cells. Monocytes were identified as Ly-6C-high cells, after excluding macrophages. Neutrophils were identified as Gr-1-high cells after excluding macrophages and monocytes (which can upregulate Gr-1 when activated). Cell quantification: Peritoneal NK cells were quantified using the LSR II, by counting the NK cells (CD122+ SSC low) in 0.5 mL of the original peritoneal 5 mL wash, and multiplying this number by 10. Other cells were quantified in a similar manner.

### NK cell cytotoxicity assay

ES-2-bearing mice were treated on days 4, 5 and 6 after ES-2 inoculation with Sin/LacZ or media. On day 7, single cell suspensions were obtained from Sin/LacZ-treated mice by washing the peritoneum with 7 ml ice-cold HBSS, or from mechanically separated spleens of mock-treated mice. NK cells were isolated from these samples using the MoFlo cell sorter (DakoCytomation, Ft. Collins, CO) by staining NK cells with anti-CD49b PE, and staining non-NK myeloid cells with anti-Gr-1 APC-eFluor® 780. NK cells were characterized and isolated as small (low FSC) and relatively rounded (low SSC) cells that were positive for CD49b, and low for Gr-1. The purity of the NK cell population was verified using FACS analysis on the LSR II, and was confirmed to be over 80% NK cells in both samples.

The cytotoxicity of NK cells was assessed using the CytoTox 96® Non-Radioactive Cytotoxicity Assay kit (Promega) according to the manufacturer's instructions. Briefly, YAC-1 cells were counted and 10,000 cells/well were added to the assay plate. NK cells were added to triplicate wells in the following ratios, (NK cells: YAC-1 cells) 5∶1, 1.25∶1, 0.312∶1, 0.078∶1 and 0.019∶1. Plates were centrifuged at 250×g for 4 minutes at 4°C and then incubated for 4 hours at 37°C. Controls for spontaneous effector cell release (NK cells alone) and spontaneous target cell release (YAC-1 cells alone) were processed in parallel. Target maximum release was also assayed in parallel. Samples were treated similarly, cells were incubated at 37°C for 3 hours then lysis solution was added and cells were incubated for an additional hour at 37°C. Samples were centrifuged at 250×g for 4 minutes at 4°C. A 50 µl volume of supernatant was transferred to a flat bottom plate and substrate mix was added. Samples were incubated for 30 minutes at room temperature and then stop solution was added. The plate was read at 490 nm using an ELx800 plate reader (Biotek Instruments, Inc.). Cytotoxicity was calculated according to the manufacturer's instructions.

### Therapeutic efficacy experiments (imaging and survival)

Treatment started on day 3 or 4 after i.p. inoculation of 1×10^6^ ES-2 cells in 0.5 mL PBS. Sin/LacZ, Sin/IL12 treatments (∼10^7^ plaque-forming units in 0.5 mL of OptiMEM I), and mock treatments (0.5 mL of OptiMEM I supplemented with 100 mg/l CaCl_2_) were administered i.p. 4 times a week for 2 weeks, for a total of 8 treatments. Therapeutic efficacy was monitored in two ways: tumor luminescence, and survival. Noninvasive bioluminescent imaging was done using the IVIS® Spectrum imaging system (Caliper Life Science, Inc.), and tumor growth was quantified using the Living Image® 3.0 software (Caliper Life Science), as previously described [18]. Survival was monitored and recorded daily.

### NK cell depletion

NK cells were depleted using either anti-CD122 (clone TM-β1; BioXcell, West Lebanon, NH), or anti-Asialo Gm1 (Wako, Dallas, Tx). In both cases, Abs were injected i.p. starting 1 day before the first Sindbis treatment, and were then injected every 2–3 days for 2 weeks (for therapeutic experiments), or less (for FACS experiments). For the anti-CD122 experiment, 20 µg Ab in 0.2 mL PBS per mouse was used in the first injection, and 10 µg Ab in 0.2 mL PBS for the subsequent injections. For the anti-Asialo Gm1 experiment, 50 µl Ab was diluted into 0.2 mL PBS per mouse. Control groups were injected with matched isotype control Ab, with PBS, or were left untreated.

### Sin/LacZ + syngeneic NK cell combination therapy

Spleens from tumor free and untreated SCID mice were mechanically separated, and NK cells were isolated from them using the MoFlo cell sorter by staining NK cells with anti-CD49b PE, and staining non-NK myeloid cells with anti-Gr-1 APC-eFluor® 780. NK cells were characterized and isolated as small (low FSC) and relatively rounded (low SSC) cells that were positive for CD49b, and low for Gr-1. The purity of the NK cell population was verified using FACS analysis on the LSR II, and was confirmed to be over 80% NK cells. 6×10^5^ NK cells in 0.2 mL HBSS were injected a single time, retro-orbitally, into ES-2-bearing mice 4 days after tumor inoculation, and 2 h before the first Sin/LacZ treatment. Control mice (mock or Sin/LacZ-treated) did not receive any NK cells. Mice were treated with Sindbis or media 4 times a week for 2 weeks, and tumor progression was determined by imaging and by monitoring survival, as in previous therapeutic efficacy experiments.

### IFNγ ELISA

For detection of peritoneal IFNγ, mice were euthanized, and their peritoneum was washed with 2 ml of cold HBSS (Mediatech). IFNγ levels in the peritoneum were determined using Mouse IFNγ ELISA Kit II (BD Biosciences) according to the manufacturer's instructions. In one experiment, the peritoneum was washed with 5 ml rather than 2 ml, so the final concentration in this experiment was normalized to the other experiments by multiplying the concentration by 2.5.

### IFNγ Block

IFNγ was blocked using XMG1.2 (BioXcell), a monoclonal anti-IFNγ Ab. 250 µg Ab in 0.2 mL PBS was injected i.p. 30 minutes before the first Sindbis treatment, and then every 2–3 days for 2 weeks (for therapeutic experiments) or less (for FACS experiments). Control mice were injected with Rat IgG isotype control Ab, or were left untreated.

### Statistics

For FACS, IVIS imaging, ELISA, and survival experiments, student *t* tests (2-tailed), 2-way ANOVA, or Kaplan-Meier log-rank test were done, using Prism® 4 for Macintosh (GraphPad Software, Inc., La Jolla, CA). Error bars shown represent the SEM.

## Results

### Sin/LacZ induces the influx of NK cells into the peritoneum of ES-2-bearing mice

We have previously shown that Sin/LacZ, a SV vector carrying the gene for β-galactosidase, targets ES-2 tumors *in vivo*, and has a therapeutic effect in SCID mice bearing these tumors [19]. In this study, we used ES-2 cells carrying firefly luciferase (ES-2/Fluc), which have previously been used to monitor tumor growth [18]. For simplicity, ES-2/Fluc cells will be referred to as ES-2 cells throughout this study.

**Figure 1:**
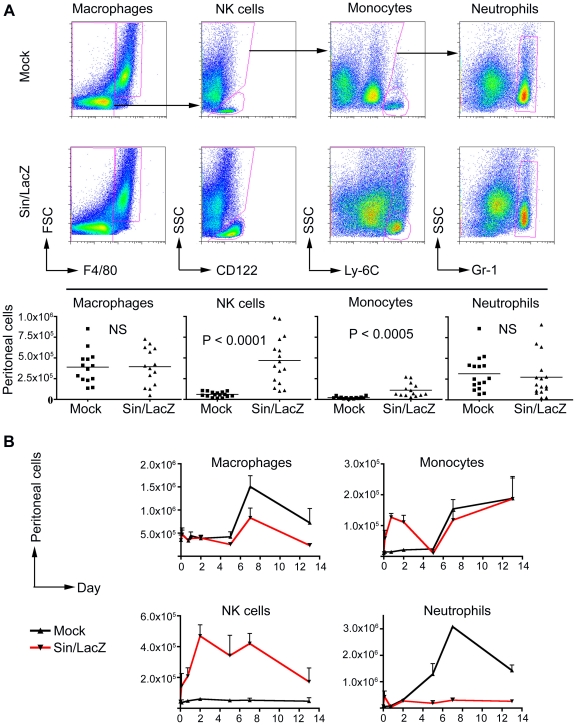
Changes in peritoneal immune cell populations in response to Sindbis/lacZ treatment in ES-2-bearing mice. (A) Top panel: ES-2 bearing mice were treated i.p. daily for two days with Sindbis/LacZ (Sin/LacZ) or media (Mock). On day 3, mice were euthanized, and cells harvested from the peritoneal cavity were analyzed by FACS as indicated. Only immune cells (CD45+ cells) were analyzed. Bottom panel: Quantification of the results from the top panel, as described in Materials and Methods; Data for A is representative of 4–5 independent experiments (n = 14–16). (B) The kinetics of changes in various peritoneal immune cell populations in ES-2 bearing mice in response to Sin/LacZ treatment are shown. Treatment started 4 days after tumor inoculation (designated day 0), and mice were treated 4 times a week. At each time point (0 h/untreated, 3 h, 18 h, 48 h, day 5, day 7, and day 13), 2–4 mice from each group were euthanized and immune cell populations were quantified as in A. Data for 0 h, 3 h, and 18 h is from one experiment; data for day 5, day 7, and day 13 is from one independent experiment per time point. Error bars represent SEM. NS  = not significant.

First, we wanted to see if SV treatment induces an immune response in SCID mice bearing ES-2 tumors. To determine the effect of SV treatment on the immune cell populations in the peritoneum, mice were treated i.p. with Sin/LacZ on days 4 and 5 after i.p. ES-2 inoculation. On day 6, the mice were euthanized, and cells harvested from the peritoneal cavity were analyzed by FACS. We found that there was a significant and reproducible influx of NK cells in response to the treatment. There was also an influx of monocytes, while the number of macrophages and neutrophils was not significantly affected by the treatment at 48 h (Fig. 1A).

Analysis of the kinetics of changes in the peritoneal immune cell populations in response to the treatment revealed that the influx of NK cells into the peritoneum started within a few hours after the first Sin/LacZ injection, and that the increased NK cell population was sustained (though slightly reduced) throughout a 2-week treatment regimen (Fig. 1B). In contrast, the increase in monocytes in response to the treatment was short-lived, with the monocyte population in Sin/LacZ-treated mice being higher than in mock-treated mice only in the first 1–2 days (Fig. 1B). Interestingly, the neutrophil population steadily increased in mock-treated mice, but was stable (and much lower) in Sin/LacZ-treated mice, in which there was only a minor early influx of neutrophils in the first hours after the first treatment (Fig. 1B). A detailed analysis of the function of these peritoneal neutrophils is beyond the scope of this study. Lastly, the total macrophage population was only slightly affected by the treatment.

In this study, we focus on NK cells, as they are the only peritoneal cell population that was consistently higher in Sin/LacZ-treated mice, and in which a higher population correlated with effective treatment. Notably, the NK cell population did not change in the Mock-treated mice (Fig. 1B), and the ES-2 tumors themselves did not induce a significant influx of NK cells into the peritoneum (data not shown). To determine if ES-2 cells were involved in the SV-induced influx of NK cells, we repeated the experiment shown in Fig 1.A in tumor-free mice. The results show that Sin/LacZ can induce the influx of NK cells even in the absence of ES-2 cells (Fig. S1). In all subsequent experiments, we used ES-2 bearing mice rather than tumor-free mice, in order to reproduce the therapeutic conditions.

### Sin/LacZ induces the activation of NK cells in the peritoneum

In order to determine if the NK cells that influx into the peritoneum in response to the treatment were activated, peritoneal NK cells from mock-treated and Sin/LacZ-treated ES-2-bearing mice were analyzed using multicolor FACS analysis. CD122+ SSC low NK cells were gated as shown in Fig 1.A, and the activation level of these cells was examined.

**Figure 2:**
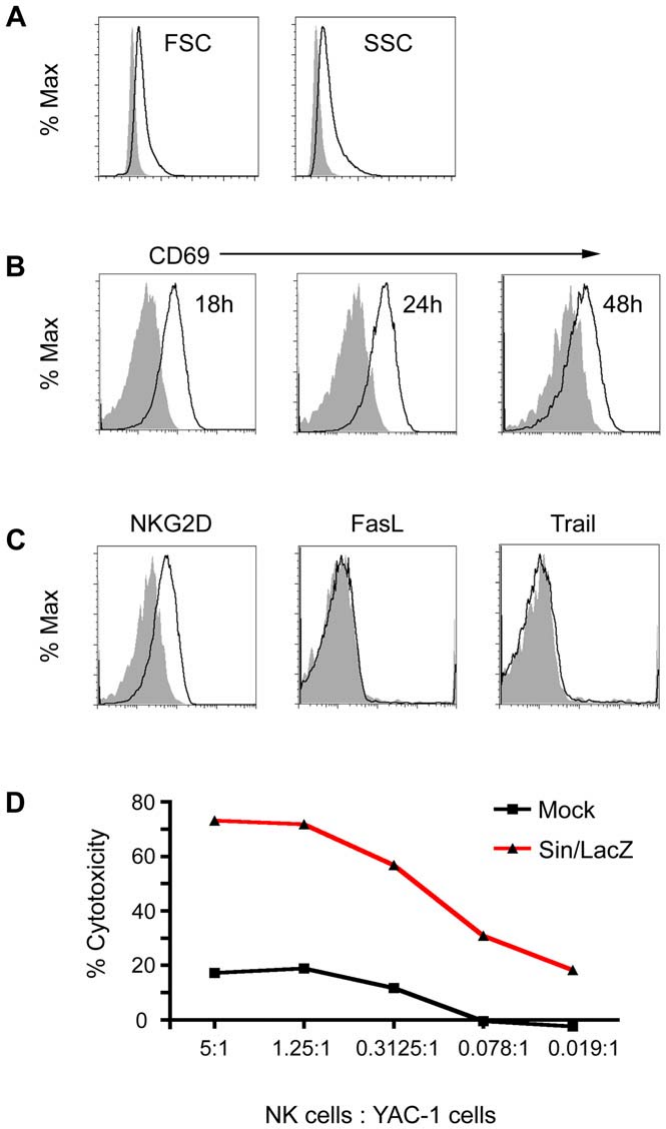
Activation of peritoneal NK cells in ES-2-bearing mice in response to Sin/LacZ treatment. (A) 48 h after the first of 2 daily i.p. Sin/LacZ treatments, the size (forward scatter; FSC) and the granularity (side scatter; SSC) of peritoneal NK cells from Sin/LacZ treated mice (black lines) was compared to peritoneal NK cells from mock-treated mice (solid grey). Data is representative of 5 independent experiments (n = 16). (B) Using the same annotation as in A, the expression of early activation marker CD69 on peritoneal NK cells from Sin/LacZ vs. mock-treated mice is shown. 18 h and 24 h samples were taken after one treatment; 48 h samples were taken after 2 treatments; each time point represents an independent experiment (n = 3 per experiment). (C) Expression of NKG2D, FasL, and Trail on NK cells 48 h after the first of 2 daily i.p. Sin/LacZ treatments; data is representative of 2 independent experiments for NKG2D (n = 5 combined), and one experiment for FasL and Trail (n = 2). (D) The cytotoxicity of peritoneal NK cells from Sin/LacZ-treated mice was determined by incubation of peritoneal NK cells from Sin/LacZ-treated mice or splenic NK cells from mock-treated mice with YAC-1 cells, as described in materials and methods; data is representative of 2 independent experiments.

We observed an increase in the size and granularity of peritoneal NK cells from Sin/LacZ-treated mice, indicating the accumulation of cytotoxic granules in these cells (Fig. 2A). We also observed that the early activation marker CD69 is expressed on peritoneal NK cells from Sin/LacZ-treated mice, an expression that peaks at around 24 h after the first treatment (Fig. 2B), and that NKG2D, a known activating receptor associated with perforin-mediated NK cell cyotoxicity [20] is upregulated in response to the treatment (Fig. 2C). However, Trail and FasL, which are upregulated on NK cells in response to some stimuli [21], [22] are not upregulated in response to Sin/LacZ treatment (Fig. 2C).

In order to confirm that peritoneal NK cells from Sin/LacZ-treated mice were activated, we tested their ability to lyse YAC-1 cells, a mouse T cell lymphoma commonly used in NK cytotoxicity assays. We found that compared to spleen NK cells from mock-treated mice, peritoneal NK cells from Sin/LacZ-treated mice had an enhanced capacity to kill YAC-1 cells (Fig. 2D). Spleen NK cells were used as the baseline rather than peritoneal NK cells because there were too few NK cells in the peritoneum of mock-treated mice.

### The efficacy of Sin/LacZ treatment is greatly reduced in SCID/beige mice

**Figure 3:**
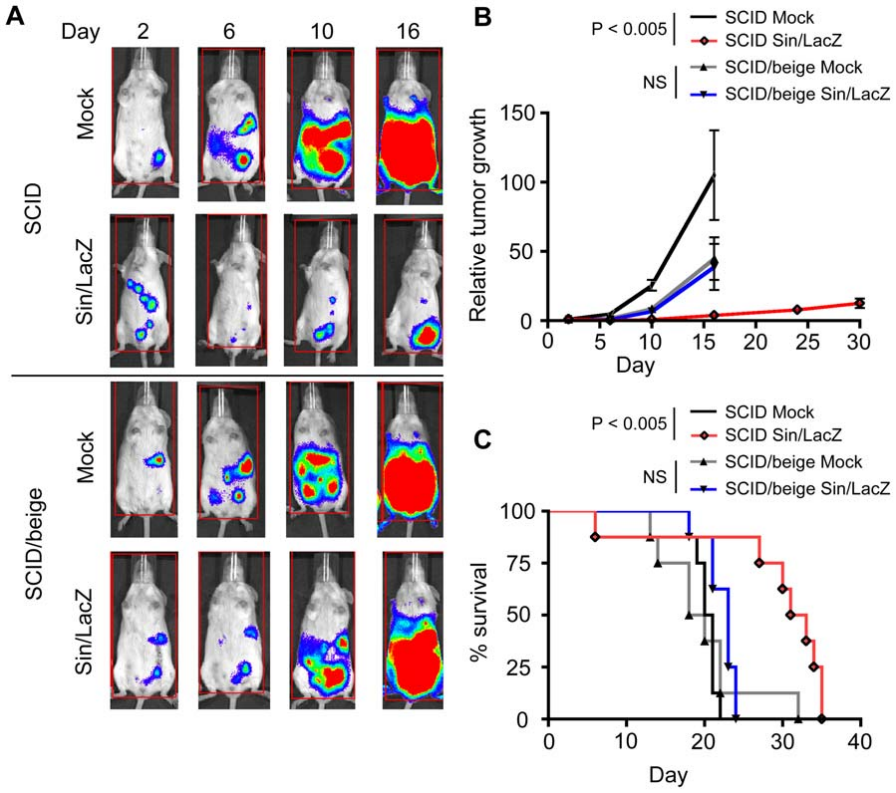
Sin/LacZ anti-ES-2 efficacy is significantly reduced in mice deficient in NK cell cytotoxicity. (A) ES-2 bearing SCID mice or SCID/beige mice were treated i.p. starting on day 3, 4 times a week, for 2 weeks, with Sin/LacZ (n = 5) or media (n = 5), and the effect of the treatment on tumor growth was monitored using IVIS. Images were taken on days 2 (one day before the treatment started), 6, 10 and 16. Representative images are shown. (B) Tumor growth from the experiment shown in A was determined by quantifying whole mouse luminescence for each individual mouse; the luminescence was then divided by the day 2 luminescence of the same mouse, to determine the fold change (relative tumor growth). Shown is the average fold change for each group (n = 5). For A and B, data from one representative of 2 independent experiments is shown. (C) Kaplan-Meier survival plots from 2 independent experiments, one of which is shown in A and B, were combined (n = 8). Error bars represent SEM.

Next we wanted to determine if the NK cells that are activated and influx into the peritoneum in response to Sin/LacZ influence the efficacy of Sin/LacZ anti-cancer treatment. To do this, ES-2-bearing SCID mice and SCID/beige mice, which have a deficiency in NK cell cytotoxicity, were treated with Sin/LacZ or with media (mock). Tumor growth was determined by measuring tumor luminescence using the IVIS imaging system, and survival was monitored daily. We found that tumors did not grow faster in mock-treated SCID/beige mice compared to mock-treated SCID mice (Fig. 3A, B and C), indicating that NK cells did not affect tumor growth in mock-treated mice. These results support the FACS data that indicated little influx or activation of NK cells in mock-treated tumor-bearing mice. Surprisingly, however, while Sin/LacZ treated SCID mice responded well to the treatment, the response to Sin/LacZ in SCID/beige mice was greatly reduced, as indicated by tumor growth (Fig. 3A and B) and survival (Fig. 3C). Indeed, in SCID/beige mice, the enhanced survival following Sin/LacZ treatment was not statistically significant, while survival was significantly enhanced in similarly treated SCID mice. These results indicate that NK cells play an important role in the efficacy of Sin/LacZ treatment in this model.

### The efficacy of Sin/LacZ treatment is greatly reduced when NK cells are depleted

**Figure 4:**
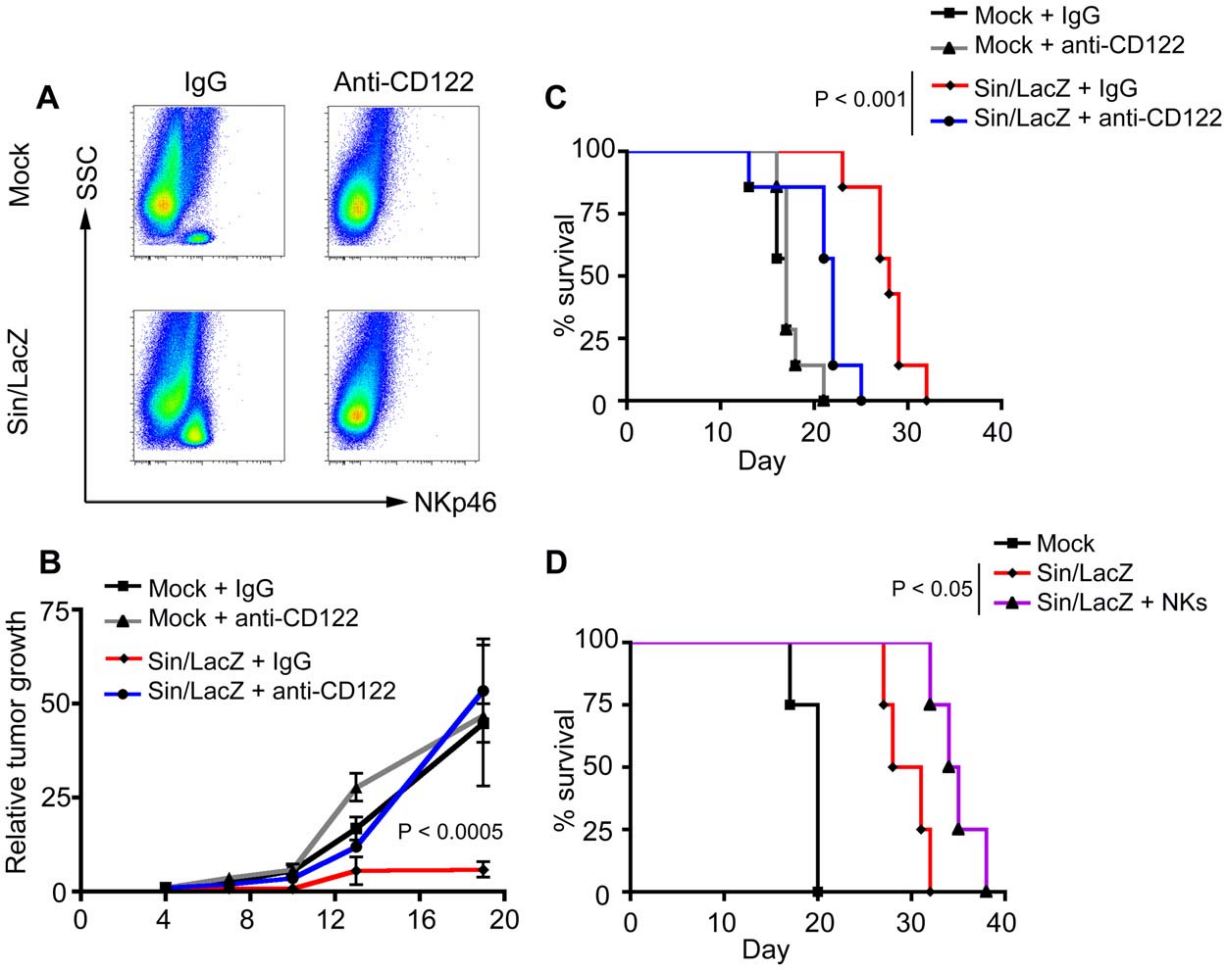
Sin/LacZ efficacy is reduced in mice depleted of NK cells, and enhanced in mice augmented with NK cells. (A) FACS analysis of cells harvested from the peritoneum of Sin/LacZ-treated and mock-treated ES-2 bearing mice that were injected with anti-CD122, or with an isotype control Ab (IgG), shows the depletion NK cells (NKp46+ SSC low) in response to the anti-CD122 treatment; data is representative of 2 independent experiments (n = 5). (B) The effect of NK cell depletion using anti-CD122 on tumor growth in Sin/LacZ and mock-treated mice is shown. The treatment scheme was similar to figure 3, but treatment and imaging started on day 4. Quantification was done as explained in figure 3; data is representative of 3 independent experiments (n = 12). (C) Kaplan-Meier survival plots from 2 independent experiments were combined (n = 7). (D) SCID mice were treated with media, Sin/LacZ or Sin/LacZ +6×10^5^ syngeneic NK cells (one retro-orbital injection 2 h before the first Sin/LacZ treatment), and their survival was plotted. Data is from one experiment (n = 4). Sin/LacZ treatments were administered 4 times a week for 2 weeks, as in previous experiments. Error bars represent SEM.

To verify that the results of the SCID/beige experiment were due to NK cell deficiency, the experiment was repeated using SCID mice that were depleted of NK cells. NK cells were depleted using a monoclonal anti-CD122 Ab, which has been used in previous studies [23]. First, to gauge the degree of NK depletion, we compared the number of NK cells in the peritoneum of anti-CD122-treated (NK depleted) vs. isotype-treated mice. NKp46 was used to identify NK cells rather than CD122, because of the possibility that CD122 would be blocked by the Ab treatment. As seen in Fig. 4A, the depletion of NK cells was effective. Notably, other cells, like monocytes and macrophages, were not depleted by anti-CD122 (Fig. S2).

For the therapeutic experiment, ES-2-bearing mice were divided into two groups. One group received anti-CD122 injections to deplete their NK cells, while the other group received control injections using a matched isotype control IgG. The groups were further divided into Sin/LacZ-treated and mock-treated groups. We observed that NK cell depletion had no significant effect on tumor growth or survival of mock-treated mice (Fig. 4B and C), again indicating that NK cells were not activated by the ES-2 tumors. In contrast, the absence of NK cells had a significant effect on Sin/LacZ-treated mice, with the efficacy of the treatment being greatly reduced in the NK cell depleted group (Fig. 4B). Survival results further confirmed this observation, as NK depletion significantly reduced the survival of Sin/LacZ treated mice (Fig. 4C). Nevertheless, Sin/LacZ treatment did appear to retain some therapeutic effect even in the absence of NK cells, indicating that Sin/LacZ efficacy is not be entirely NK cell dependent.

To confirm that the depletion of NK cells, rather than an artifact of using anti-CD122, is responsible for these results, we also used anti-asialo GM1 Abs to deplete NK cells [24], and obtained similar results (data not shown). Taken together, three separate models of NK cell deficiency have demonstrated that the efficacy of Sin/LacZ treatment in this model is largely, though not completely, NK cell dependent.

### The efficacy of Sin/LacZ treatment is enhanced when syngeneic NK cells are pre-injected into the mice

We have shown that Sin/LacZ can activate NK cells against ES-2 tumors in SCID mice (Fig. 3 and 4A, B and C). Next, we wanted to determine if the therapeutic effect of Sin/LacZ could be enhanced by the addition of syngeneic NK cells. To test this, NK cells were isolated from the spleens of tumor-free untreated mice, and injected into mice retro-orbitally 2 hours before the first Sin/LacZ treatment. Tumor growth was monitored using IVIS, and was not significantly affected by the addition of NK cells (data not shown). However, the survival of mice treated with Sin/LacZ + NK cells was improved compared to mice treated with Sin/LacZ alone (Fig. 4D).

### The efficacy of Sin/IL12 treatment is also NK cell dependent

**Figure 5:**
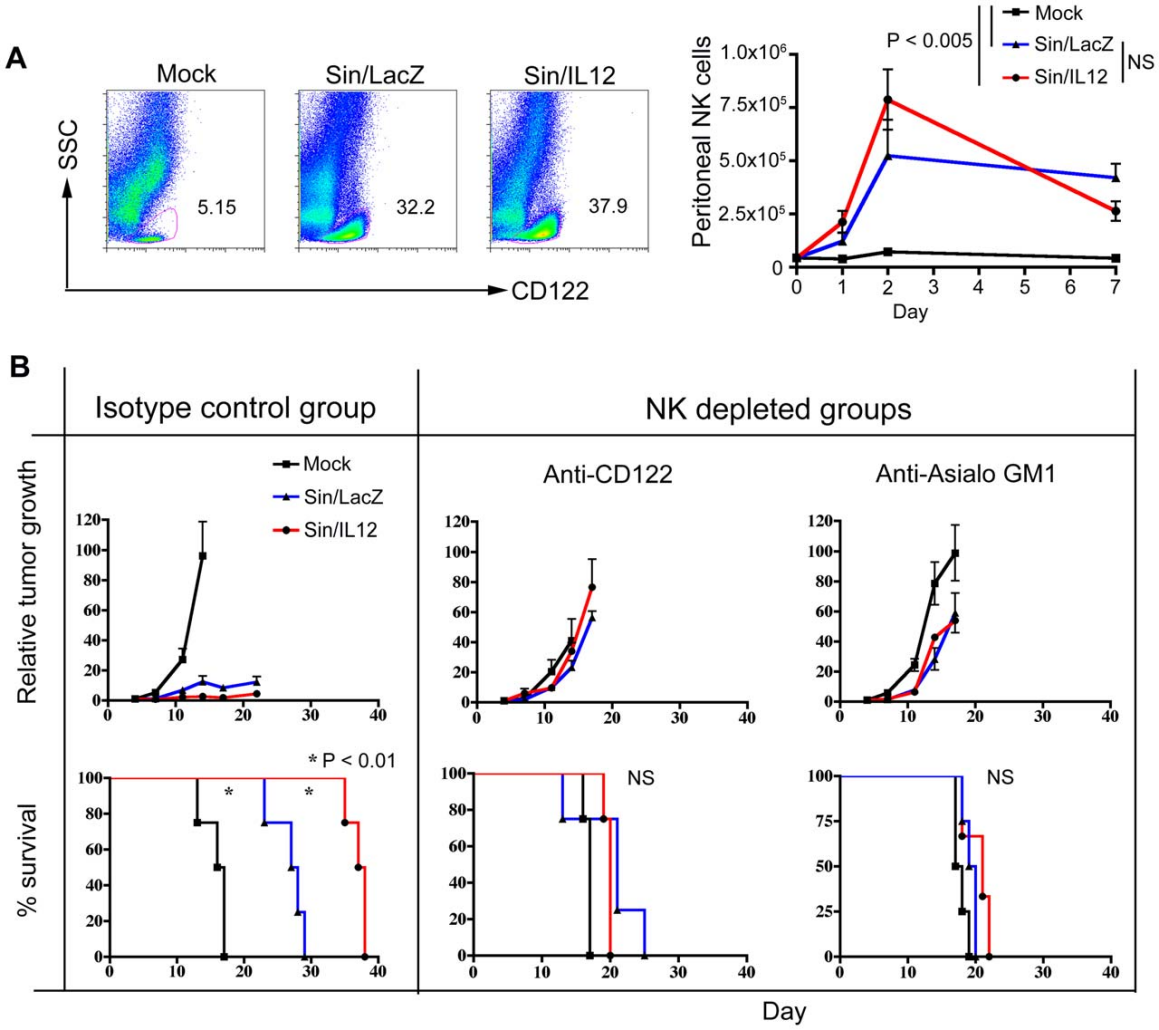
The efficacy of Sin/IL12 treatment is also dependent on NK cells. (A) Left panel: FACS analysis of peritoneal samples from mock, Sin/LacZ, or Sin/IL12-treated ES-2-bearing mice shows the percentage of NK cells (CD122+ SSC low) within the CD45+ (immune cell) gate 48 h after the first of two daily Sin/LacZ or Sin/IL12 treatments. Data is representative of one experiment (n = 4). Right panel: kinetics of the change in peritoneal NK cell numbers in response to the treatment. Four time points (days 0/untreated, 1, 2, and 7) are shown (n = 2–4 for each time point). (B) The experiment shown in figure 4B and C was repeated, adding Sin/IL12-treated mice. NK cells were depleted with either anti-CD122 (middle panels) or anti-Asialo GM1 (right panels). Tumor growth and survival were monitored, quantified and plotted as in figure 4. Data is representative of one experiment (n = 3–4, as shown). Error bars represent SEM.

Our previous results have demonstrated that SV vectors carrying IL12 have greater therapeutic efficacy than Sin/LacZ in the ES-2 tumor model [19]. In order to determine the role of NK cells in this increased efficacy, the experiments shown in Fig. 1 and 4 were repeated with Sin/IL12-treated mice in addition to Sin/LacZ–treated mice. FACS analysis of cells harvested by peritoneal lavage from Sin/IL12-treated mice showed that Sin/IL12 induced a similar influx of NK cells as Sin/LacZ (Fig. 5A). NK cell depletion experiments using anti-CD122 and anti-asialo GM1 demonstrated that the therapeutic effect of both Sin/LacZ and Sin/IL12 was largely abrogated in the absence of NK cells (Fig. 5B). Notably, the therapeutic enhancement obtained from Sin/IL12 compared to Sin/LacZ (Fig 5B; left panel) was also essentially eliminated in the absence of NK cells (right panels), demonstrating that the enhanced efficacy of Sin/IL12 was also NK cell-dependent.

It should be noted, that in SCID/beige mice, Sin/IL12 retained a moderate therapeutic effect (Fig. S3A), and FACS analysis revealed that there was a strong influx of NK cells in SCID/beige mice treated with Sin/IL12 (Fig. S3B). As mentioned, SCID/beige mice have a deficiency in NK cell cytotoxicity. However, they still have NK cells, which retain some regulatory functions, like the ability to secrete IFNγ in response to IL12.

### The enhanced therapeutic effect from Sin/IL12 is IFNγ-dependent

**Figure 6:**
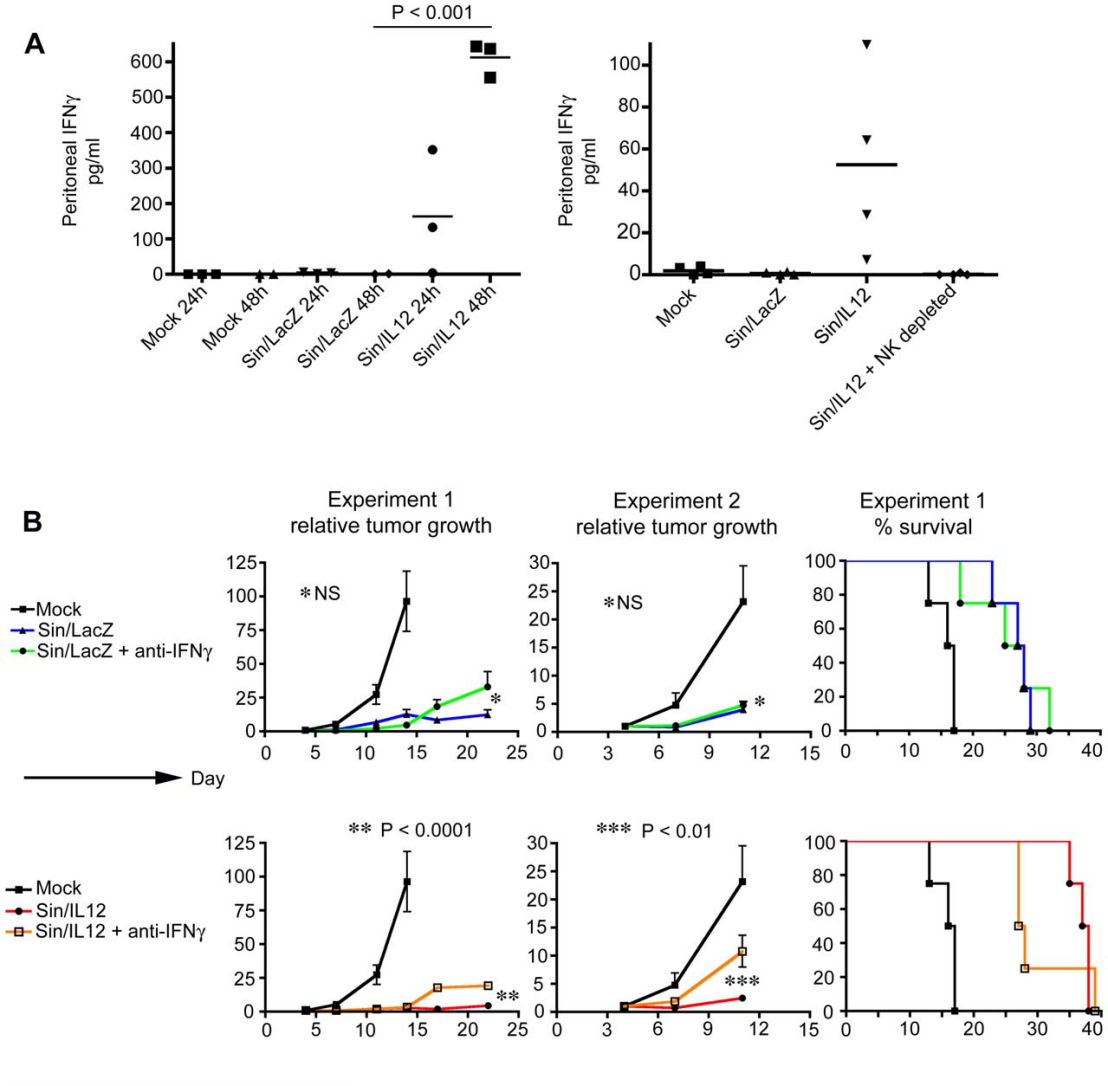
The enhanced therapeutic effect of Sin/IL12 compared to Sin/LacZ is IFNγ dependent. (A) Left panel: ES-2 bearing mice were treated daily for two days with Sin/LacZ, Sin/IL12, or media. After 24 h (one treatment), or 48 h (two treatments), mice were euthanized, and their peritoneum was washed with HBSS. IFNγ levels in the peritoneal washes was determined by ELISA. Data is from one experiment per time point (n = 2–3 as indicated). Right panel: The experiment was repeated, and an additional group in which NK cells were depleted using anti-CD122 was added. Data is from one experiment (n = 4). (B) ES-2 bearing mice were treated with media, Sin/LacZ, Sin/IL12, Sin/LacZ + anti-IFNγ (to block IFNγ activity) or Sin/IL12 + anti-IFNγ. The effect of the IFNγ blockade was determined by monitoring tumor growth and mouse survival, as explained in previous figures. Data is representative of 2 independent experiments, as shown (n = 4 for experiment 1; n = 2–3 for experiment 2). Error bars represent SEM.

IL12 can induce the secretion of IFNγ from NK cells [25]. Since we determined that the enhanced efficacy of Sin/IL12 is NK cell dependent (Fig. 5B), we hypothesized that IFNγ secretion by NK cells might be involved in this therapeutic enhancement. To test this, we measured IFNγ levels in the peritoneum of Sin/LacZ, Sin/IL12, and mock-treated ES-2-bearing mice. We determined that both mock-treated and Sin/LacZ-treated mice have almost no peritoneal IFNγ 24 h or 48 h after treatment was started. In contrast, Sin/IL12-treated mice had high levels of IFNγ in the peritoneum at 48 h (Fig. 6A, left panel). To verify that the source of IFNγ was Sin/IL12-activated NK cells, we repeated the IFNγ assay in Sin/IL12-treated mice that were depleted of NK cells, and confirmed that in the absence of NK cells, Sin/IL12 did not induce the secretion of IFNγ in the peritoneum (Fig. 6A, right panel).

In order to determine if Sin/LacZ and/or Sin/IL12 treatment efficacy is dependent on IFNγ, we repeated the therapeutic experiments described above, and added additional groups in which IFNγ activity was blocked with a monoclonal Ab. As expected, blocking IFNγ had no effect on Sin/LacZ treatment. However, it did reduce the efficacy of Sin/IL12 treatment (Fig. 6B). Survival results indicated that in the absence of IFNγ, Sin/IL12 treatment is comparable to Sin/LacZ treatment (Fig. 6B, right panel), further suggesting that IFNγ is responsible for the increased efficacy obtained from Sin/IL12 treatment.

### Sin/IL12 induces the NK cell-dependent IFNγ-dependent upregulation of MHC class II expression on peritoneal macrophages

**Figure 7:**
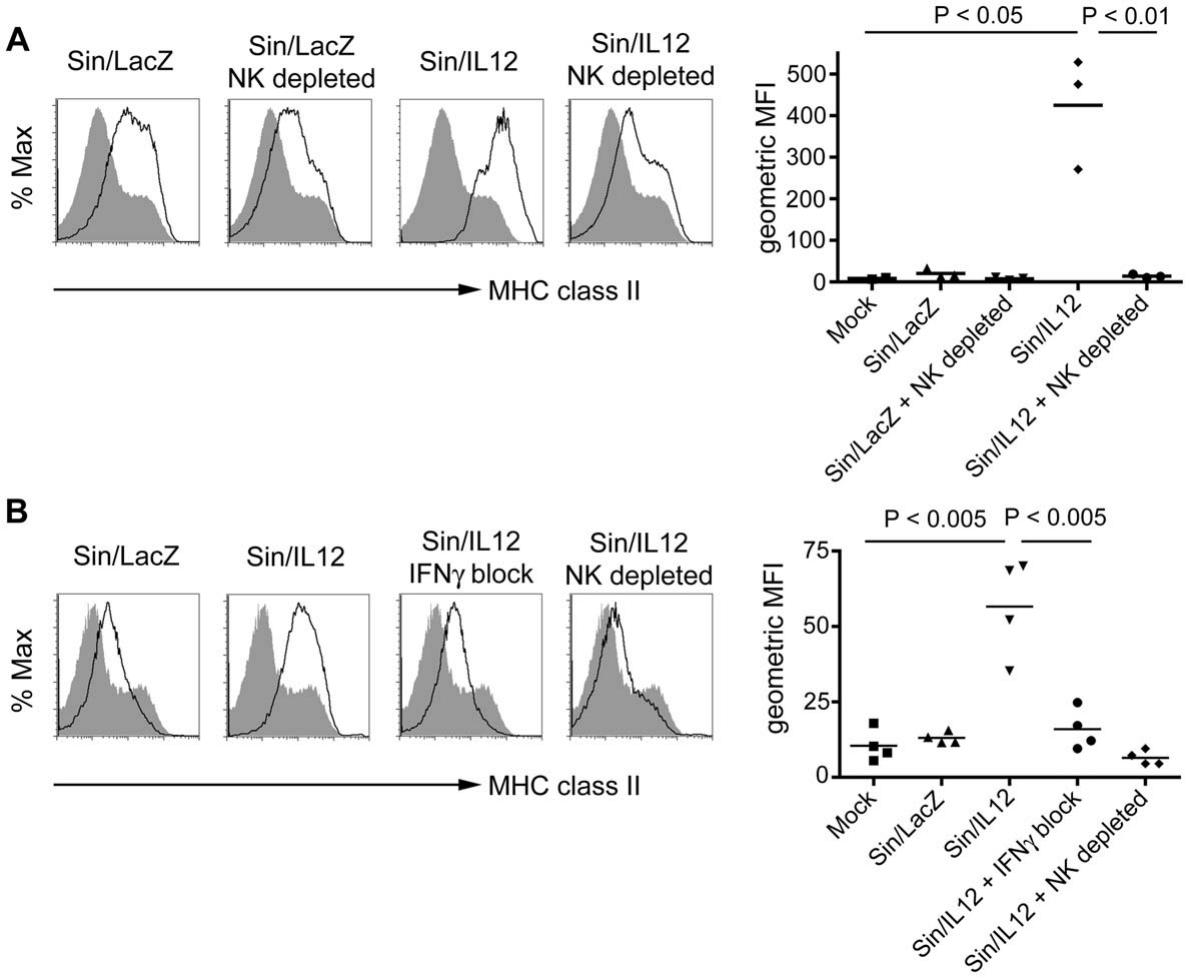
Sin/IL12 induces the NK cell-dependent, IFNγ-dependent expression of MHC class II on peritoneal macrophages. (A) ES-2 bearing mice were treated for one week (4 treatments) with media, Sin/LacZ, Sin/LacZ + anti-CD122 (NK cell depleted), Sin/IL12, or Sin/IL12 + anti-CD122, after which mice were euthanized, and the expression of MHC class II on peritoneal macrophages (F4/80+ FSC high cells) was analyzed by FACS (left panel), and quantified (right panel) (n = 2–3 as shown). Black lines represent NK cells from the various Sindbis-treated mice, and the solid grey curve represents NK cells from mock-treated mice. (B) ES-2 bearing mice were treated daily for two days with media, Sin/LacZ, Sin/IL12, Sin/IL12 + anti-IFNγ (IFNγ block), or Sin/IL12 + anti-CD122 (NK cell depleted). MHC expression was analyzed and quantified as in A. Data is from one experiment (n = 4).

Because IL12 is a known Th1 cytokine [26], we wanted to determine if Sin/IL12 could influence macrophages in the tumor environment by shifting them towards an M1 anti-cancer phenotype. We also wanted to determine if NK cells are involved in the activation of peritoneal macrophages. To do this, we repeated the Sin/LacZ and Sin/IL12 NK cell depletion experiment, but terminated the experiment after 1 week, at which point the mice were sacrificed and cells harvested from the peritoneum were analyzed. To determine the expression of the M1 marker MHC class II on macrophages, we stained peritoneal cells with Abs against the macrophage marker F4/80 and MHC class II. We determined that Sin/LacZ induced only a minor increase in MHC II expression on macrophages, but Sin/IL12 induced a much greater increase (Fig. 7A). Notably, depleting NK cells largely inhibited the increase in MHC II (Fig. 7A).

IFNγ has been shown to induce the expression of MHC class II on macrophages in some models [27]. Because we determined that NK cells secrete IFNγ in response to Sin/IL12 (Fig. 6A), and because the expression of MHC class II on peritoneal macrophages in response to Sin/IL12 was NK cell dependent (Fig. 7A), we wanted to determine if IFNγ is responsible for the increase in MHC class II. To do this, we treated tumor-bearing mice with media (mock), Sin/LacZ, Sin/IL12, Sin/IL12 + anti- IFNγ (to block IFNγ activity), or Sin/IL12 + anti-CD122 (to deplete NK cells). After two consecutive treatments (one per day), we sacrificed the mice (on the third day), and analyzed the peritoneal macrophage population. We determined that the Sin/IL12-dependent induction of MHC class II expression on macrophages was abolished in absence of IFNγ or NK cells (Fig. 7B).

## Discussion

Clinical studies have demonstrated that OV anti-cancer efficacy in mouse models doesn't always translate well to human patients. One possible reason for this discrepancy is an incomplete understanding of the mechanism behind the successful results in mice. Most OVs were originally identified by their ability to preferentially kill cancer cells *in vitro* [6]. As a result, the therapeutic efficacy of OV treatments *in vivo* has traditionally been attributed to direct viral oncolysis. Advances in animal imaging techniques have enabled researchers to demonstrate tumor-specific targeting of human and mouse tumors by a few OVs [18], [28], further supporting the direct viral oncolysis hypothesis. However, in some cases, effective OV cancer therapy did not require progressive replication and oncolysis [29], or was achieved even in tumor models that are not efficiently lysed by the OV *in vitro* [30], pointing to a possible indirect anti-cancer effect. In the last few years, studies in immunocompetent mouse models have demonstrated an important role for immune cell activation in several OV therapies [2], [9], [10]. Recently, innate immune responses in xenograft OV models have also been reported [30], [31], [32], however, the therapeutic relevance of these responses has not been characterized. The goal of this study was to determine if innate immune responses, and in particular NK cells, are involved in the therapeutic efficacy of SV therapy in a well-established xenograft model for ovarian cancer.

NK cell cytotoxicity is activated by the interactions between activating receptors on the NK cells with stress ligands on target cells, and by the absence of interactions between inhibitory receptors on the NK cells with MHC class I molecules on target cells [12]. Both virally infected cells and cancer cells can potentially upregulate stress ligands [12], [33], and downregulate MHC class I [12], [34], marking them for NK cell mediated lysis. However, for an effective NK response to occur, peripheral NK cells need to be activated and recruited to the site of infection/tumor. Moreover, in the case of cancer, tumor-induced immunosuppression often needs to be overcome. The current study provides proof of concept that (i) Sin/LacZ treatment can activate NK cells and recruit them to the tumor environment, where their cytolytic function is redirected against ES-2 tumor cells, and (ii) Sin/IL12 treatment can modulate the regulatory functions of NK cells, resulting in the induction of anti-cancer M1 macrophages, which can potentially reverse cancer-induced immunosuppression.

The immune response to viral infections is triggered by the recognition of viral pathogen-associated molecular patterns (PAMPs) [35] or by danger signals generated by virally induced tissue damage [36], and results in the recruitment and activation of a large number of effector cells at the site of infection. Cancer cells lack PAMPs, and often don't induce a strong danger signal [37], resulting in poor immune cell activation. In this study, we show that SV-activated NK cells are recruited to the peritoneum of SCID mice, where they target and kill i.p. growing ES-2 tumors. The influx of NK cells into the peritoneum, and their activation as demonstrated by an increase in size and granularity, are independent of the ES-2 tumor cells, and also occur in tumor-free mice that were treated with SV. Furthermore, SV-activated peritoneal NK cells can also lyse Yac-1 cells, suggesting a general activation of NK cell cytotoxicity rather than a response against the xenograft. Indeed, in the absence of SV, ES-2 tumors do not induce the activation or the influx of NK cells.

Nevertheless, unlike naturally occurring tumors, xenografts are foreign tissues, and might therefore be particularly susceptible to SV-activated NK cells. This reveals a general limitation of this mouse model, and might explain the high efficacy observed in other OV xenograft models in which the role of NK cells has not yet been examined. For example, it has previously been reported that replication-competent Sindbis has an anti-cancer effect in a peritoneal dissemination model for ovarian cancer [38]. In that study, Sindbis was able to inhibit the abdominal swelling that results from ascites formation in nude mice bearing human OMC-3 tumors. These effects are similar to what we have observed in our ES-2 model using replication deficient SV vectors (Fig 3A, Day 16, and additional unpublished results). However, in the absence of functional NK cells, the inhibition of abdominal swelling is greatly reduced, indicating that SV-activated NK cells are largely responsible for this therapeutic effect in our model. Notably, we have previously shown that SV-dependent inhibition of abdominal swelling also occurred in immunocompetent mice bearing syngeneic ovarian tumors [19]. Additional studies are needed to determine the role of SV-activated NK cells in that model.

Another limitation of SCID mouse models is the absence of regulatory T cells and other factors that can generate an immunosuppressive tumor environment. It is not clear if SV treatment alone can overcome this suppression, which can inhibit NK cell function [39]. One way to potentially increase the ability of SV-activated NK cells to work in an immunosuppressive environment is by engineering a Sindbis vector that can deliver specific immune modulators to the tumor environment. IL12 is a potent cytokine, which can have numerous effects on tumor growth, including activation of NK cells, and the induction of IFNγ [40]. SV vectors carrying IL12 have been shown to have an enhanced therapeutic effect, but the mechanism has not been established [18]. Here we show that Sin/IL12-activated NK cells, but not Sin/LacZ-activated NK cells, secrete IFNγ, and that IFNγ plays a critical role in the enhanced therapeutic effect obtained from Sin/IL12.

IFNγ can exert anti-cancer effects in several different ways [41]. Ovarian cancer requires angiogenesis to grow, so the antiangiogenic effects of IFNγ [42] are one possible therapeutic mechanism. Another appealing function of IFNγ is its influence on tumor-associated macrophages, which are often immunosuppressive [43]. As a key Th1 cytokine, IFNγ can shift the tumor-associated macrophage population towards the M1 anti-cancer phenotype. In one report, IFNγ and double stranded RNA, a viral PAMP, have been shown to individually and additively activate macrophages [44]. In our model, Sin/IL12, but not Sin/LacZ or Sin/IL12 + IFNγ blockade, induced the expression of the M1 activation marker MHC class II on peritoneal macrophages. Notably, this induction was NK cell dependent, demonstrating that SV vectors can activate the regulatory function of NK cells as well as their cytotoxicity. NK cells have also been reported to provide IFNγ for Th1 priming in an immunocompetent mouse model [45]. Further studies are needed to determine the effect of Sin/IL12 therapy on T cell activity.

In addition to their effect on immune cells, some OVs might influence tumor cells directly, for example, by inducing the expression of stress markers, thereby making them more susceptible to effector cell mediated lysis [46], [47]. However, if OV infection by itself results in the death of infected cells, as is often the case, then effector cell mediated lysis of infected tumor cells is redundant and not therapeutic. SV infection results in the induction of apoptosis in mammalian cells [48], [49], including ES-2 cells (our unpublished results). We have demonstrated that NK cells are responsible for most of the therapeutic efficacy of SV, therefore it seems likely that SV-activated NK cells can also target uninfected ES-2 cells. One of the limitations of using replication defective vectors, which are safer, is low infectivity, because the virus cannot replicate inside the tumor. However, our results suggest that SV treatment results in a strong NK cell-mediated bystander effect, demonstrating that high infectivity might not always be required for effective OV therapy.

Ironically, another consequence of using replication competent vectors is that their propagation could be inhibited by NK cell-mediated lysis of infected cells, as has been recently reported [50]. In that study, in which vesicular stomatitis virus vectors were used for cancer therapy in immunocompetent rats bearing hepatocellular carcinoma, the inhibition of NK and NKT cells was therapeutically beneficial. These results are in stark contrast to other studies, including our current study, in which NK cell inhibition is detrimental. Such discrepancies between OV studies underscore the complexity of the interactions involved [2], [5]. Ultimately, the role of NK cell activation in OV therapy will depend on the properties of the OV and on the particular cancer being treated, in addition to any underlying differences in NK cell function between patients.

**Figure 8:**
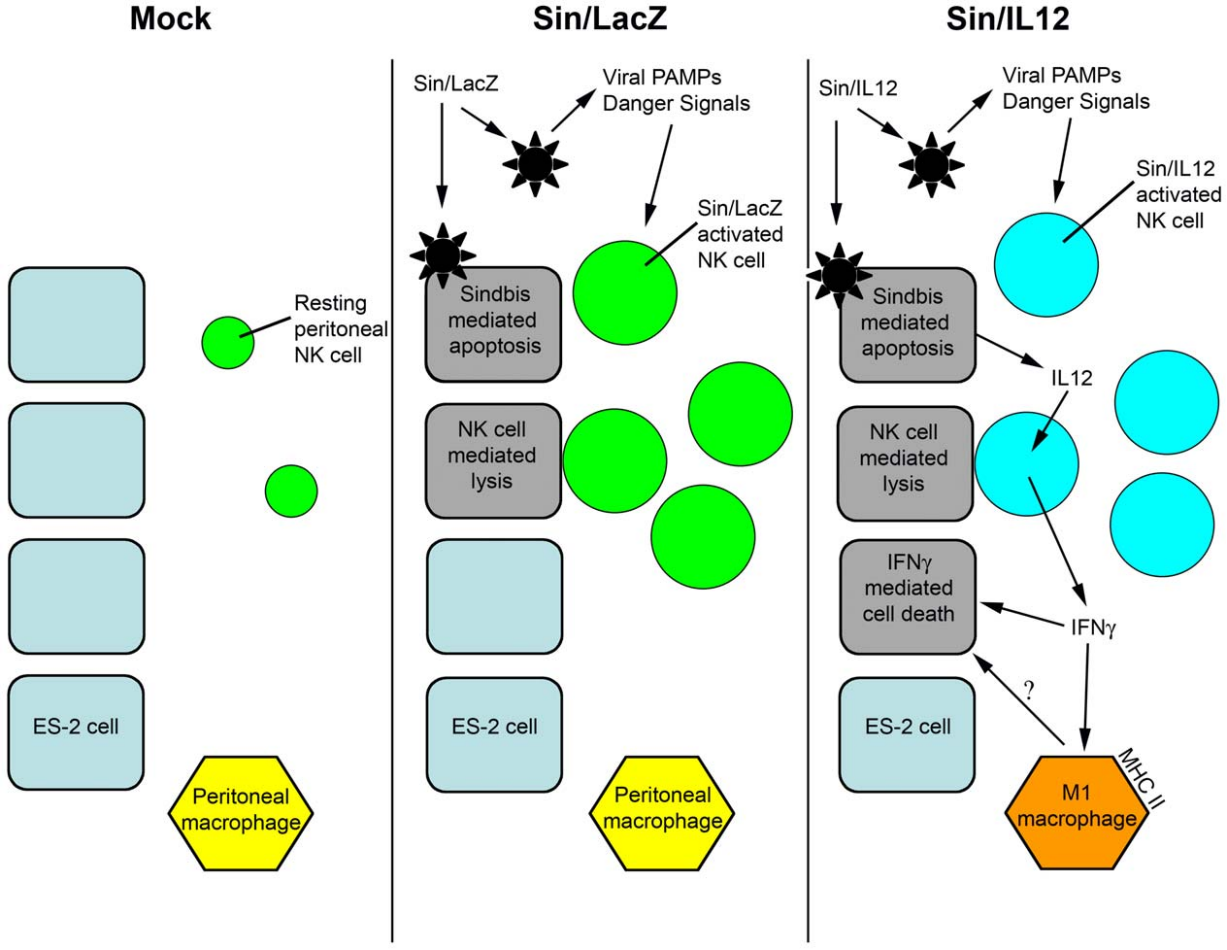
Model for the activation of NK cells by Sindbis vectors. Left panel: ES-2 cells are not susceptible to resting NK cells, and cannot activate or recruit NK cells. Middle panel: Sin/LacZ targets ES-2 cells, inducing apoptosis of infected cells. At the same time, viral PAMPs and/or virus-induced danger signals lead to the recruitment and activation of NK cells at the tumor site. Sin/LacZ-activated NK cells target and lyse ES-2 cells, including uninfected cells (bystander effect). Right panel: Sin/IL12 activates and recruits NK cells by the same mechanism as Sin/LacZ, but in addition, leads to the secretion of IL12 from ES-2 cells, which induces the secretion of IFNγ from NK cells, resulting in the activation of peritoneal macrophages and an enhanced therapeutic effect.

Based on the results of this study, we propose a model for NK cell activation by SV vectors (Fig. 8). We have previously shown that treatment with Sin/LacZ (middle panel) results in the infection of ES-2 cells [19]. However, the signals initiating NK cell recruitment and activation originate in host cells, because these events also occur in tumor-free mice. NK cell recruitment and activation likely involves the secretion of danger signals or exposure to viral PAMPs, but additional studies are needed to elucidate the precise mechanism. Sin/IL12 treatment also induces the recruitment and activation of NK cells, but in addition, it results in the production and secretion of IL12 from ES-2 cells. IL12, in turn, induces the secretion of IFNγ by SV-activated NK cells in the peritoneum, resulting in an enhanced therapeutic effect, and in the upregulation of the M1 marker MHC class II on peritoneal macrophages (right panel). We have not yet determined if the activation of macrophages is responsible for the therapeutic effect induced by Sin/IL12-dependent IFNγ. However, the induction of M1 macrophages is a key step in reducing the immunosuppressive influence of the tumor environment, and is therefore a central goal of immunotherapy.

Cancer is the result of the accumulation of multiple defects in cell regulation and behavior, making it difficult to combat the disease with simple therapeutic regimens. Consequently, novel and combinatorial treatments are sought that can target tumor growth from different directions, reducing the likelihood of treatment resistance. By better understanding the role of immune activation by SV vectors, we can better predict possible positive (or negative) interactions between SV treatment and other therapies. For example, we can attempt to induce a synergistic effect by combining SV with other immunotherapeutic agents, like cytokines or autologous NK cells. Alternatively, we can seek a non-overlapping additive effect by activating immune cells with SV, while killing tumor cells directly with chemotherapy and SV-induced apoptosis. The immunosuppressive functions of some chemotherapeutic agents also need to be considered, to avoid possible antagonistic effects. However, ‘targeted immunosuppression’ could in theory be beneficial, if it can inhibit the immune response to the virus (and therefore increase direct viral oncolysis) without significantly suppressing the immune response against the tumor.

The significance of this study is that it provides a proof-of-principle for NK cell activation by SV vectors. Additional studies are needed to compliment these results. The SCID model used here demonstrates that NK cell activation by SV vectors is possible, and therefore presents us with a novel goal for enhancing OV therapy using SV vectors. Future studies with immunocompetent models will determine whether or not T cells or B cells interfere or support the activation of NK cells by SV vectors, and in the process, may provide us with valuable information as to what is needed for effective NK cell activation in these models. *In vitro* studies using human immune cells will also complement our current study, revealing any interspecies differences between humans and mice, and laying the ground for future clinical studies.

## Supporting Information

Figure S1 **Sin/LacZ induces the influx of NK cells into the peritoneum, and an increase in peritoneal NK cell granularity in tumor-free mice.** The experiment shown in Fig 1.A was repeated in tumor-free mice. Top panel: representative FACS plots from 2 independent experiments (n = 6). Bottom panel: quantification of the cell populations. Error bars represent SEM.
(TIF)

Figure S2 **The effect of anti-CD122 injections on the peritoneal immune cell populations in Sin/LacZ-treated ES-2-bearing SCID mice.** Mice were treated with Sin/LacZ for one week (for a total of 4 injections), and were injected with anti-CD122 or no Ab every 2–3 days starting one day before the first Sin/LacZ treatment. Top panel: representative FACS plots from one experiment (n = 3). Bottom panel: quantification of the cell populations.
(TIF)

Figure S3 **The effect of Sin/IL12 treatment on SCID/beige mice bearing ES-2 tumors.** (A) ES-2 bearing SCID/beige mice were treated i.p. starting day 3, 4 times a week, for 2 weeks, with Sin/LacZ, Sin/IL12 or media, and the effect of the treatment on tumor growth was monitored using IVIS as in figure 3 (n = 5). B. Top: Mice were treated with media, Sin/LacZ or Sin/IL12 on days 4, 5, 8, 9, and 10 after ES-2 inoculation, and were euthanized on day 12, after which peritoneal cells were harvested and analyzed by FACS. Bottom: Quantification of the peritoneal NK cell populations from the top panel. Data is from one experiment (n = 2).
(TIF)

## References

[1] Vaha-Koskela MJ, Heikkila JE, Hinkkanen AE (2007). Oncolytic viruses in cancer therapy.. Cancer Lett.

[2] Prestwich RJ, Errington F, Diaz RM, Pandha HS, Harrington KJ (2009). The case of oncolytic viruses versus the immune system: waiting on the judgment of Solomon.. Hum Gene Ther.

[3] Bramson JL, Hitt M, Addison CL, Muller WJ, Gauldie J (1996). Direct intratumoral injection of an adenovirus expressing interleukin-12 induces regression and long-lasting immunity that is associated with highly localized expression of interleukin-12.. Hum Gene Ther.

[4] Leonard JP, Sherman ML, Fisher GL, Buchanan LJ, Larsen G (1997). Effects of single-dose interleukin-12 exposure on interleukin-12-associated toxicity and interferon-gamma production.. Blood.

[5] Hirasawa K, Nishikawa SG, Norman KL, Coffey MC, Thompson BG (2003). Systemic reovirus therapy of metastatic cancer in immune-competent mice.. Cancer Res.

[6] Martuza RL, Malick A, Markert JM, Ruffner KL, Coen DM (1991). Experimental therapy of human glioma by means of a genetically engineered virus mutant.. Science.

[7] Grote D, Russell SJ, Cornu TI, Cattaneo R, Vile R (2001). Live attenuated measles virus induces regression of human lymphoma xenografts in immunodeficient mice.. Blood.

[8] Vaha-Koskela MJ, Kallio JP, Jansson LC, Heikkila JE, Zakhartchenko VA (2006). Oncolytic capacity of attenuated replicative semliki forest virus in human melanoma xenografts in severe combined immunodeficient mice.. Cancer Res.

[9] Toda M, Rabkin SD, Kojima H, Martuza RL (1999). Herpes simplex virus as an in situ cancer vaccine for the induction of specific anti-tumor immunity.. Hum Gene Ther.

[10] Diaz RM, Galivo F, Kottke T, Wongthida P, Qiao J (2007). Oncolytic immunovirotherapy for melanoma using vesicular stomatitis virus.. Cancer Res.

[11] Hicks AM, Riedlinger G, Willingham MC, Alexander-Miller MA, Von Kap-Herr C (2006). Transferable anticancer innate immunity in spontaneous regression/complete resistance mice.. Proc Natl Acad Sci U S A.

[12] Vivier E, Tomasello E, Baratin M, Walzer T, Ugolini S (2008). Functions of natural killer cells.. Nat Immunol.

[13] Wang KS, Kuhn RJ, Strauss EG, Ou S, Strauss JH (1992). High-affinity laminin receptor is a receptor for Sindbis virus in mammalian cells.. J Virol.

[14] Tseng JC, Zheng Y, Yee H, Levy DE, Meruelo D (2007). Restricted tissue tropism and acquired resistance to Sindbis viral vector expression in the absence of innate and adaptive immunity.. Gene Ther.

[15] Bredenbeek PJ, Frolov I, Rice CM, Schlesinger S (1993). Sindbis virus expression vectors: packaging of RNA replicons by using defective helper RNAs.. J Virol.

[16] Tseng JC, Zanzonico PB, Levin B, Finn R, Larson SM (2006). Tumor-specific in vivo transfection with HSV-1 thymidine kinase gene using a Sindbis viral vector as a basis for prodrug ganciclovir activation and PET.. J Nucl Med.

[17] Tseng JC, Levin B, Hirano T, Yee H, Pampeno C (2002). In vivo antitumor activity of Sindbis viral vectors.. J Natl Cancer Inst.

[18] Tseng JC, Levin B, Hurtado A, Yee H, Perez de Castro I (2004). Systemic tumor targeting and killing by Sindbis viral vectors.. Nat Biotechnol.

[19] Tseng JC, Hurtado A, Yee H, Levin B, Boivin C (2004). Using sindbis viral vectors for specific detection and suppression of advanced ovarian cancer in animal models.. Cancer Res.

[20] Bauer S, Groh V, Wu J, Steinle A, Phillips JH (1999). Activation of NK cells and T cells by NKG2D, a receptor for stress-inducible MICA.. Science.

[21] Kayagaki N, Yamaguchi N, Nakayama M, Takeda K, Akiba H (1999). Expression and function of TNF-related apoptosis-inducing ligand on murine activated NK cells.. J Immunol.

[22] Zamai L, Ahmad M, Bennett IM, Azzoni L, Alnemri ES (1998). Natural killer (NK) cell-mediated cytotoxicity: differential use of TRAIL and Fas ligand by immature and mature primary human NK cells.. J Exp Med.

[23] Tanaka T, Kitamura F, Nagasaka Y, Kuida K, Suwa H (1993). Selective long-term elimination of natural killer cells in vivo by an anti-interleukin 2 receptor beta chain monoclonal antibody in mice.. J Exp Med.

[24] Habu S, Fukui H, Shimamura K, Kasai M, Nagai Y (1981). In vivo effects of anti-asialo GM1. I. Reduction of NK activity and enhancement of transplanted tumor growth in nude mice.. J Immunol.

[25] Chan SH, Perussia B, Gupta JW, Kobayashi M, Pospisil M (1991). Induction of interferon gamma production by natural killer cell stimulatory factor: characterization of the responder cells and synergy with other inducers.. J Exp Med.

[26] Manetti R, Parronchi P, Giudizi MG, Piccinni MP, Maggi E (1993). Natural killer cell stimulatory factor (interleukin 12 [IL-12]) induces T helper type 1 (Th1)-specific immune responses and inhibits the development of IL-4-producing Th cells.. J Exp Med.

[27] King DP, Jones PP (1983). Induction of Ia and H-2 antigens on a macrophage cell line by immune interferon.. J Immunol.

[28] Shafren DR, Au GG, Nguyen T, Newcombe NG, Haley ES (2004). Systemic therapy of malignant human melanoma tumors by a common cold-producing enterovirus, coxsackievirus a21.. Clin Cancer Res.

[29] Galivo F, Diaz RM, Wongthida P, Thompson J, Kottke T (2009). Single-cycle viral gene expression, rather than progressive replication and oncolysis, is required for VSV therapy of B16 melanoma.. Gene Ther.

[30] Yu YA, Galanis C, Woo Y, Chen N, Zhang Q (2009). Regression of human pancreatic tumor xenografts in mice after a single systemic injection of recombinant vaccinia virus GLV-1h68.. Mol Cancer Ther.

[31] Worschech A, Haddad D, Stroncek DF, Wang E, Marincola FM (2009). The immunologic aspects of poxvirus oncolytic therapy.. Cancer Immunol Immunother.

[32] Worschech A, Chen N, Yu YA, Zhang Q, Pos Z (2009). Systemic treatment of xenografts with vaccinia virus GLV-1h68 reveals the immunologic facet of oncolytic therapy.. BMC Genomics.

[33] Cerwenka A, Baron JL, Lanier LL (2001). Ectopic expression of retinoic acid early inducible-1 gene (RAE-1) permits natural killer cell-mediated rejection of a MHC class I-bearing tumor in vivo.. Proc Natl Acad Sci U S A.

[34] Karre K, Ljunggren HG, Piontek G, Kiessling R (1986). Selective rejection of H-2-deficient lymphoma variants suggests alternative immune defence strategy.. Nature.

[35] Janeway CA (1989). Approaching the asymptote? Evolution and revolution in immunology.. Cold Spring Harb Symp Quant Biol 54 Pt.

[36] Matzinger P (1994). Tolerance, danger, and the extended family.. Annu Rev Immunol.

[37] Matzinger P (2002). The danger model: a renewed sense of self.. Science.

[38] Unno Y, Shino Y, Kondo F, Igarashi N, Wang G (2005). Oncolytic viral therapy for cervical and ovarian cancer cells by Sindbis virus AR339 strain.. Clin Cancer Res.

[39] Smyth MJ, Teng MW, Swann J, Kyparissoudis K, Godfrey DI (2006). CD4+CD25+ T regulatory cells suppress NK cell-mediated immunotherapy of cancer.. J Immunol.

[40] Colombo MP, Trinchieri G (2002). Interleukin-12 in anti-tumor immunity and immunotherapy.. Cytokine Growth Factor Rev.

[41] Ikeda H, Old LJ, Schreiber RD (2002). The roles of IFN gamma in protection against tumor development and cancer immunoediting.. Cytokine Growth Factor Rev.

[42] Voest EE, Kenyon BM, O'Reilly MS, Truitt G, D'Amato RJ (1995). Inhibition of angiogenesis in vivo by interleukin 12.. J Natl Cancer Inst.

[43] Elgert KD, Alleva DG, Mullins DW (1998). Tumor-induced immune dysfunction: the macrophage connection.. J Leukoc Biol.

[44] Heitmeier MR, Scarim AL, Corbett JA (1998). Double-stranded RNA-induced inducible nitric-oxide synthase expression and interleukin-1 release by murine macrophages requires NF-kappaB activation.. J Biol Chem.

[45] Martin-Fontecha A, Thomsen LL, Brett S, Gerard C, Lipp M (2004). Induced recruitment of NK cells to lymph nodes provides IFN-gamma for T(H)1 priming.. Nat Immunol.

[46] Routes JM, Ryan S, Morris K, Takaki R, Cerwenka A (2005). Adenovirus serotype 5 E1A sensitizes tumor cells to NKG2D-dependent NK cell lysis and tumor rejection.. J Exp Med.

[47] Ogbomo H, Michaelis M, Geiler J, van Rikxoort M, Muster T (2010). Tumor cells infected with oncolytic influenza A virus prime natural killer cells for lysis of resistant tumor cells.. Med Microbiol Immunol.

[48] Levine B, Huang Q, Isaacs JT, Reed JC, Griffin DE (1993). Conversion of lytic to persistent alphavirus infection by the bcl-2 cellular oncogene.. Nature.

[49] Venticinque L, Meruelo D (2010). Sindbis viral vector induced apoptosis requires translational inhibition and signaling through Mcl-1 and Bak.. Mol Cancer.

[50] Altomonte J, Wu L, Meseck M, Chen L, Ebert O (2009). Enhanced oncolytic potency of vesicular stomatitis virus through vector-mediated inhibition of NK and NKT cells.. Cancer Gene Ther.

