# Supplementary figures and images for: Activation of Cytotoxic and Regulatory Functions of NK Cells by Sindbis Viral Vectors
Source: PLoS One. 2011 Jun 2;6(6):e20598. doi: 10.1371/journal.pone.0020598 (PMC3107224; doi:10.1371/journal.pone.0020598)

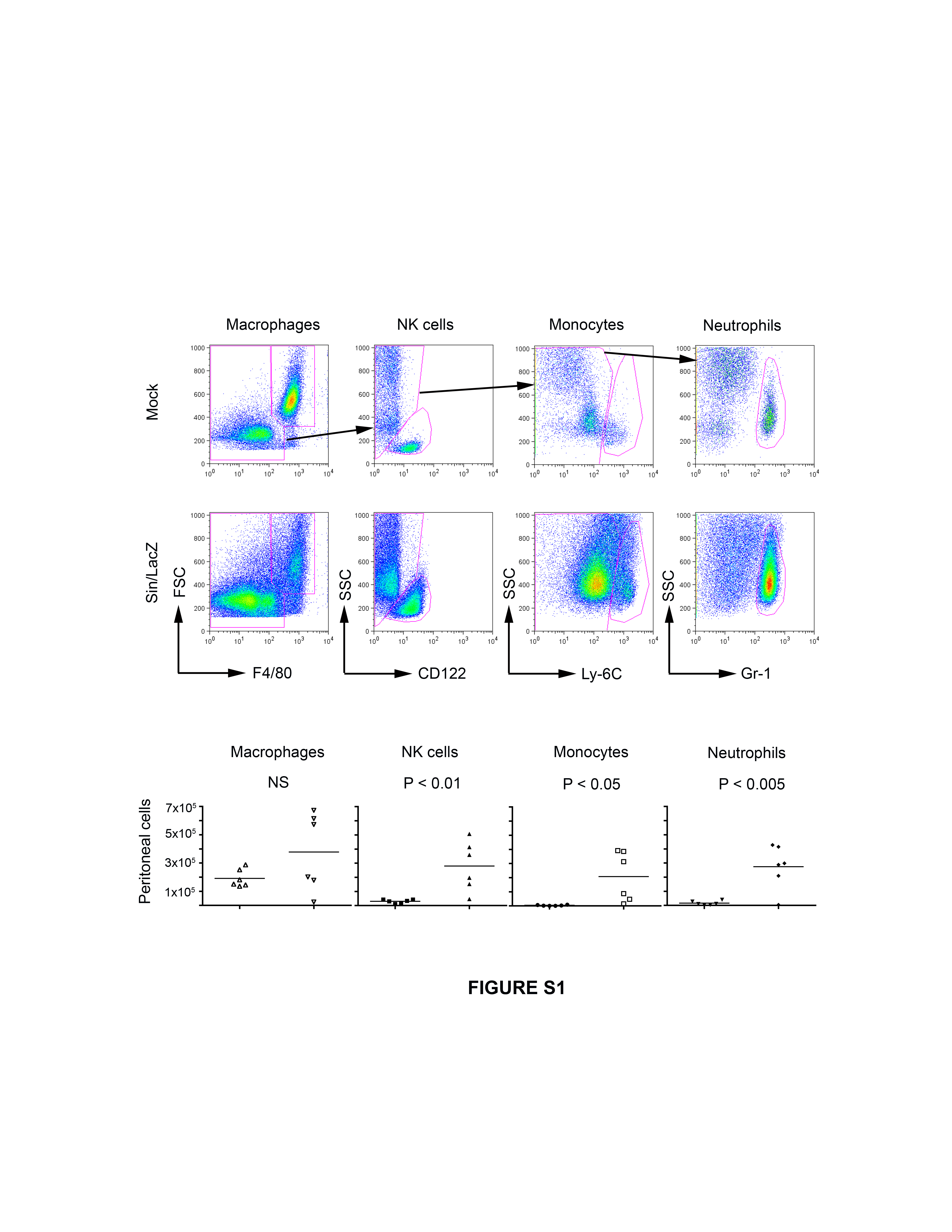

Supplement: Figure S1 — Sin/LacZ induces the influx of NK cells into the peritoneum, and an increase in peritoneal NK cell granularity in tumor-free mice. The experiment shown in Fig 1.A was repeated in tumor-free mice. Top panel: representative FACS plots from 2 independent experiments (n = 6). Bottom panel: quantification of the cell populations. Error bars represent SEM. (TIF) [file pone.0020598.s001.tif]

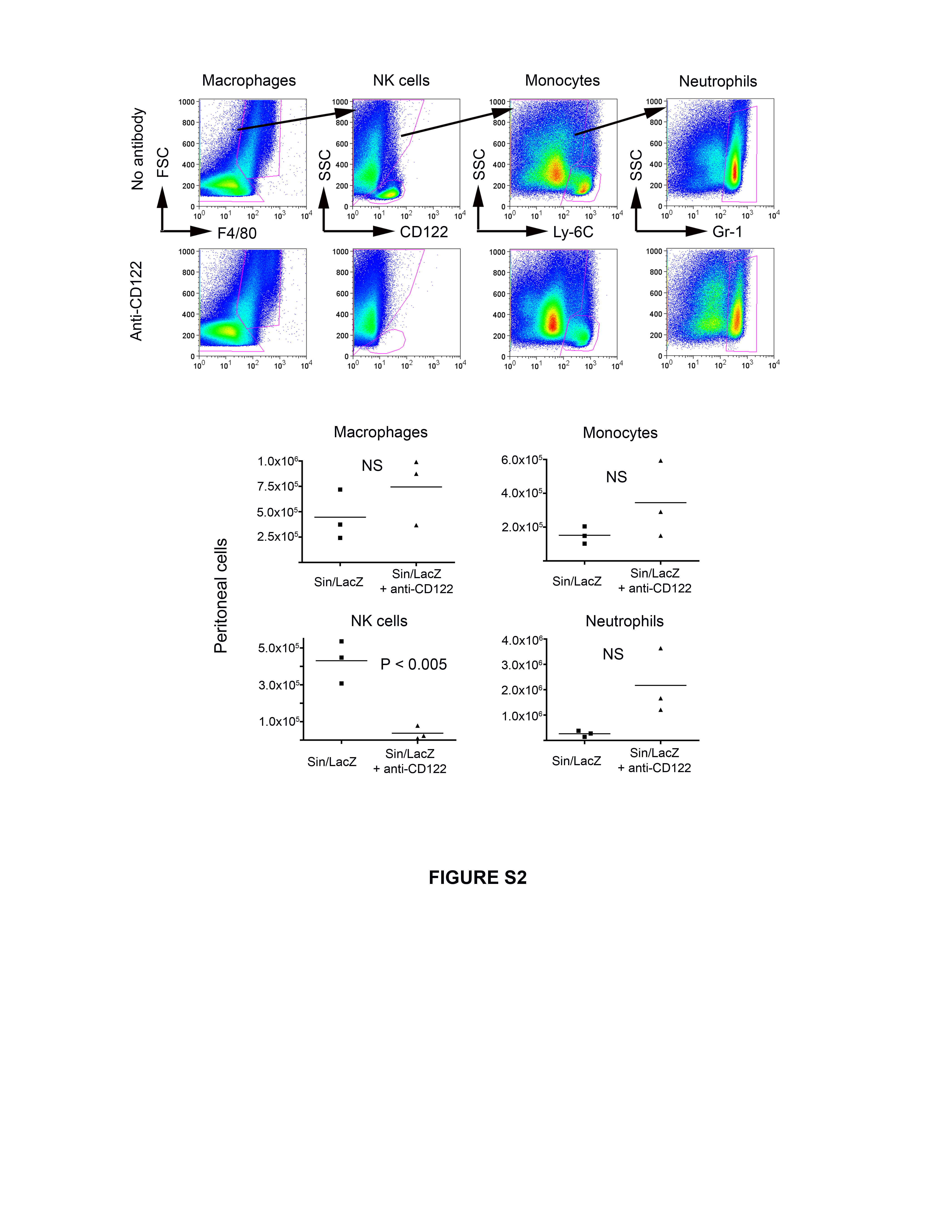

Supplement: Figure S2 — The effect of anti-CD122 injections on the peritoneal immune cell populations in Sin/LacZ-treated ES-2-bearing SCID mice. Mice were treated with Sin/LacZ for one week (for a total of 4 injections), and were injected with anti-CD122 or no Ab every 2–3 days starting one day before the first Sin/LacZ treatment. Top panel: representative FACS plots from one experiment (n = 3). Bottom panel: quantification of the cell populations. (TIF) [file pone.0020598.s002.tif]

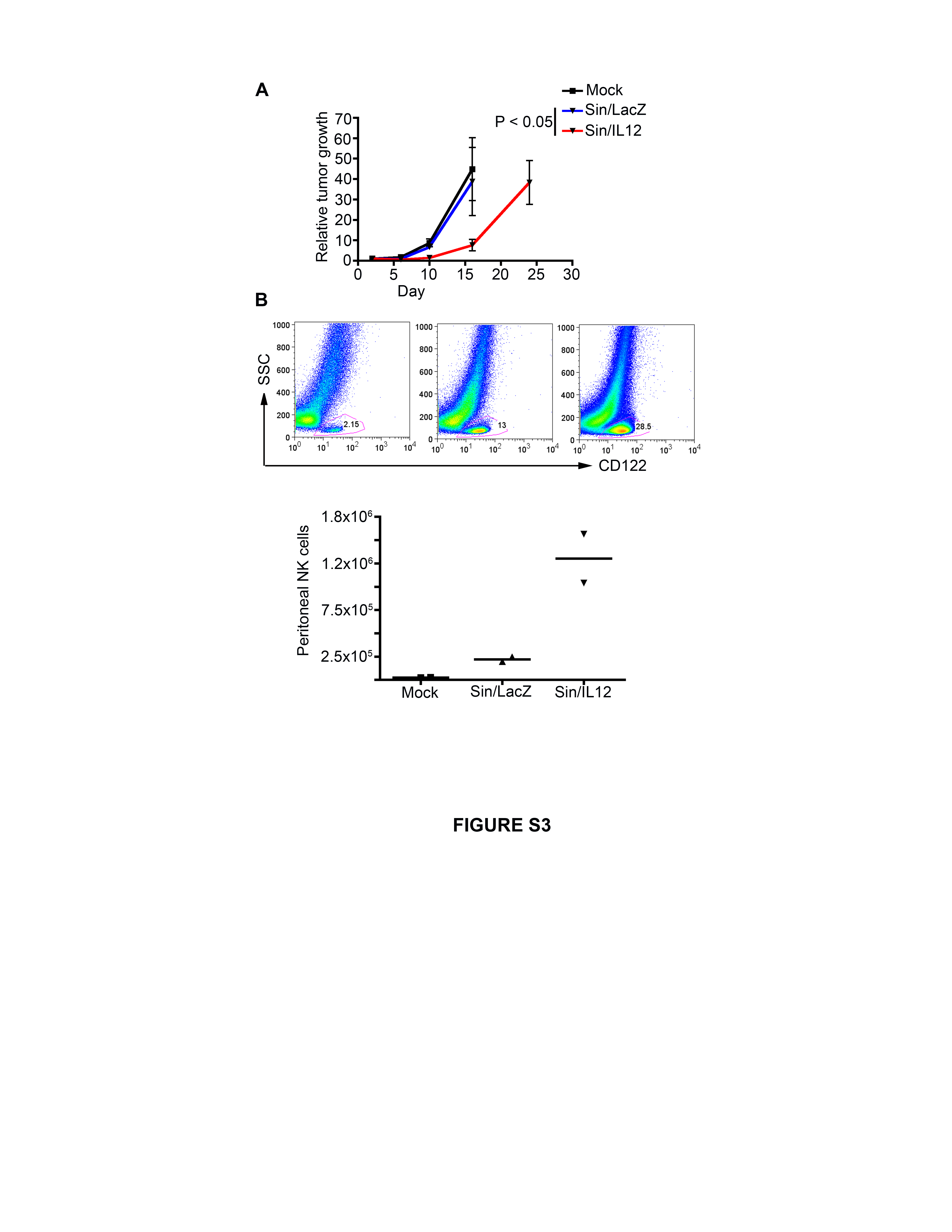

Supplement: Figure S3 — The effect of Sin/IL12 treatment on SCID/beige mice bearing ES-2 tumors. (A) ES-2 bearing SCID/beige mice were treated i.p. starting day 3, 4 times a week, for 2 weeks, with Sin/LacZ, Sin/IL12 or media, and the effect of the treatment on tumor growth was monitored using IVIS as in figure 3 (n = 5). B. Top: Mice were treated with media, Sin/LacZ or Sin/IL12 on days 4, 5, 8, 9, and 10 after ES-2 inoculation, and were euthanized on day 12, after which peritoneal cells were harvested and analyzed by FACS. Bottom: Quantification of the peritoneal NK cell populations from the top panel. Data is from one experiment (n = 2). (TIF) [file pone.0020598.s003.tif]
